# Cerebellar-cerebral circuits functional connectivity in patients with cognitive impairment after basal ganglia stroke: a pilot study

**DOI:** 10.3389/fnagi.2025.1478891

**Published:** 2025-01-30

**Authors:** Lijun Zuo, Xinlong Lan, Yijun Zhou, Hao Liu, Yang Hu, Yongjun Wang, Tao Liu, Zixiao Li

**Affiliations:** ^1^Department of Neurology, Beijing Tiantan Hospital, Capital Medical University, Beijing, China; ^2^Beijing Advanced Innovation Center for Biomedical Engineering, School of Biological Science and Medical Engineering, Beihang University, Beijing, China; ^3^National Clinical Research Center for Neurological Diseases, Beijing Tiantan Hospital, Capital Medical University, Beijing, China

**Keywords:** stroke, cognitive impairment, functional connectivity, cerebellum, cerebellar-cerebral circuits

## Abstract

**Introduction:**

This study aims to assess the pattern of functional connectivity (FC) between cerebellar subregions, the basal ganglia (BG), and the cortex, and explore the relationship between FC patterns and cognitive function after stroke with BG infarcts.

**Methods:**

A total of 39 stroke patients and 29 healthy controls were recruited. Four cerebellar seed points were selected, and the FC of each seed point with other voxels in the whole brain was calculated. FC and cognitive performance were compared between the two groups, and their correlations were analyzed.

**Results:**

Stroke patients exhibited increased FC between the bilateral cerebellum IX and BG (particularly the head of the caudate nucleus), which was positively correlated with episodic memory, visuospatial ability, and attention. Increased FC was also observed between the right cerebellum Crus I/II and BG, as well as the bilateral cerebellum VI and BG, correlating positively with episodic memory. Conversely, decreased FC was identified between the bilateral cerebellum IX and the right caudal cuneus, which negatively correlated with episodic memory, language, and attention but positively correlated with executive function. Additionally, increased FC between the bilateral cerebellum VI and the bilateral inferior parietal lobule was associated with improvements in episodic memory, language, and attention. Decreased FC was observed between the right cerebellum VI and the left insula, as well as between the right cerebellum Crus I/II and the left insula, which negatively correlated with episodic memory.

**Discussion:**

The enhanced FC between the cerebellum and BG, along with the reorganization of new neural circuits involving the cerebellar cortex, may contribute to cognitive recovery following stroke. These changes may represent compensatory mechanisms of the cerebellum in response to stroke injury.

## 1 Introduction

Poststroke cognitive impairment (PSCI) is one of the common consequences of ischemic stroke, affecting attention, memory, visuospatial dysfunction, or other aspects (Zhang and Bi, 2020). The basal ganglia (BG) is the most common site for acute ischemic stroke (AIS). The BG infarction is associated with an increased risk of cognitive impairment, such as executive function (Papagno and Trojano, 2018; Silveri, 2021). For BG, the caudate and putamen are the main input nuclei, receiving axons from the cortex, such as the dorsolateral prefrontal cortex (DLPFC) (Grahn et al., 2008). The substantia nigra and globus pallidus are the main output nuclei. The cortico-basal ganglia-thalamo-cortical circuits are largely conceived as parallel loops, including limbic, associative, and sensorimotor loop separately (Aoki et al., 2019; DeLong and Wichmann, 2010; Li et al., 2022). The associative loop comprises the head of caudate and most rostral putamen, which receive input from the DLPFC, presupplementary motor area and posterior parietal cortex (Parent, 1990; Parent and Hazrati, 1995). The DLPFC is one of key regions of the central executive network (CEN) which involved processing information and maintaining cognitive function. Therefore, studies have reported that repetitive transcranial magnetic stimulation (rTMS) stimulation of DLPFC has a good effect on improvement of memory dysfunction (Quinn et al., 2018; Camacho-Conde et al., 2023). The TMS coil can stimulate up to 5 cm into the brain structures. Therefore, most of the targets of TMS for PSCI with BG infarcts are focused on cognitive-related regions of the cerebral cortex.

The BG are reported to be closely associated with the cerebellum. Both of BG and cerebellum receive input from, and send output to the cortex regulating movement and cognitive function (Caligiore et al., 2017; Sokolov et al., 2017). The cerebellum could be divided into 10 subregions signified by Roman numerals, from the anterior (regions I–V) to the posterior (regions VI–X) (Buckner et al., 2011). The cerebellum IX showed strong functional connectivity (FC) with the Default Mode Network (DMN), and the cerebellum Crus I and Crus II showed strong FC with the fronto-parietal network, and reciprocal connections with the DLPFC (Kelly and Strick, 2003). The cerebellum VI was correlated with the SN (Salience network). The cerebellar circuits are activated simultaneously during various cognitive processing such as working memory (Chen and Desmond, 2005), executive function (Chen and Desmond, 2005), and language (Ackermann et al., 2007). A previous study found that the FC of the right dentate nucleus and right cerebellum was associated with cognitive behaviors in MSA patients (Yang et al., 2020). All the above data indicated that different cerebellar areas had connections with different neural networks involved in cognition. The cerebellum and the BG project to the same motor areas, as well as the premotor, temporal, and parietal cortex (Koziol et al., 2014). They are components of a highly integrated cortical–subcortical functional network that supports motor control and encompasses cognitive function (Schmahmann, 2019). It is called the basal-ganglia-cerebellar-cortical network (Silveri, 2021).

Nevertheless, the FC patterns among the cerebellum, BG and cerebral cortex and their associations with different cognitive domains in PSCI patients have not been fully characterized and the underlying mechanisms of PSCI with BG infarcts remain unclear. Exploring the FC between the cerebral cortex and the cerebellum using rs-fMRI may help to explore new potential targets of cognitive recovery and intervention in PSCI patients with BG infarcts. The cerebellum, therefore, might be a new target of rTMS for PSCI with BG infarcts.

The present study was designed to explore the FCs between the cerebellum and the cerebral cortex and associations with different cognitive domains in ischemic stroke patients and controls, and to explore neuroimaging targets for early intervention of PSCI with BG infarcts.

## 2 Materials and methods

### 2.1 PSCI patients

A total of 39 mild ischemic stroke patients with first-ever BG infarcts were enrolled, along with 29 age, gender, and educational level-matched healthy controls (HC). The stroke patients were chosen from the Department of Neurology in Beijing Tiantan hospital between August 2015 and November 2016.

Major inclusion criteria included: (1) age 35–65 years; (2) presented with ischemic stroke within 7 days; (3) the infarction lesions were BG regions; (4) with a National Institute of Health Stroke Scale (NIHSS) score ≤ 3.

The exclusion criteria included: (1) stroke mimics, such as seizures; (2) illiteracy; (3) obvious demyelination or silent infarction on magnetic resonance imaging (MRI) image; (4) psychiatric diseases with a Hamilton Depression Rating Score (HAMD) ≥ 17; (5) delirium or pre-stroke dementia with a score of Informant Questionnaire on Cognitive Decline in the Elderly (IQCODE) > 3.38 (Jorm and Jacomb, 1989); (6) Other factors interfering with cognitive evaluation, e.g., severe aphasia [National Institutes of Health Stroke Scale (NIHSS) 9 > 2], consciousness disorders (NIHSS 1a > 1 or 1b > 1), Hearing or vision disorders, severe unilateral neglect or dyslexia.

### 2.2 Control subjects

The HCs had no previous history of neurological disease, mental disorders, myelination, or lacune on MRI. All participants provided written informed consent.

### 2.3 Neuropsychological assessment and clinical function

All participants were assessed for their cognition and clinical features within 10 days of admission. The Montreal Cognitive Assessment (MoCA)-Beijing was used to evaluate global cognitive function (Yu et al., 2012). One point was added to the total MoCA score for those with 12 years of education or less (Nasreddine et al., 2005). The neuropsychological test battery examined five cognitive domains (Table 1). Activities of daily living were assessed by the basic ADL scale (BADL) and instrumental ADL (IADL) scale. Emotion was assessed by the Hamilton Depression Scale (HAMD) (Hamilton, 1960).

**Table 1:** Cognitive tests comprising each of the domains assessed.

Cognitive domains	Task	Index
Episodic memory		
	Auditory Verbal Learning Test	Total score; delayed recall score
	Rey-Osterrieth Complex Figure Test (RCFT) memory	Delayed recall score
Language		
	Animal Fluency Test (AFT)	Total numbers animals generated
	Boston Naming Test (BNT, 30-item)	Total numbers correct with no cue
Visuospatial ability		
	RCFT-copy	Total copy score
Attention		
	Symbol Digital Modality Test (SDMT)	Total numbers correct
	Chinese modified version of Trail Making Test (TMT)-A	
Executive function	Chinese modified version of Trail Making Test (TMT)-B-TMT-A	Time (s)
	Chinese modified version of Stroop Color-Word Test (CWT)-Color	Time(s)

The time interval between the cognitive assessment and MRI scanning was 12 hour. The *Z* score for the cognitive tests was calculated as a score that fell within the distribution of scores for the HCs. Cognitive impairment was set as a score of 1.5 standard deviations (SD) below the published norms. The diagnosis for identifying cognitive impairment was based on the Diagnostic and Statistical Manual of Mental Disorders, 4th edition (DSM-4).

### 2.4 Image acquisition

MRI images were acquired on a 3.0 T Prisma MRI scanner (Siemens Healthcare, Erlangen, Germany) from the Department of Functional Neuroimaging, Beijing Institute of Neurosurgery, Capital Medical University. The rs-fMRI data for all subjects was obtained by an echo-planar-imaging (EPI) sequence with repetition time (TR) = 2,500 ms, echotime (TE) = 30 ms, flip angle = 90°, voxel size = 2.86 × 2.86 × 3 mm^3^, image matrix = 70 × 70 × 43, 200 volumes. High-resolution T1-weighted scans were acquired using the following parameters: TR = 2,300 ms, TE = 2.3 ms, flip angle = 8°, voxel size = 1 × 1 × 1 mm^3^, image matrix = 256 × 256 × 176.

### 2.5 Image data preprocessing

The DPABI software was used to preprocess fMRI data^1^ and implemented in the MATLAB platform (The MathWorks, Natick, MA, United States). The first 10 time points were not analyzed to allow for the stabilization of the BOLD signal and magnetization. The data is rearranged and corrected in time and space dimensions. Motion correction of each participant’s data based on SPM12 parameters was performed to calculate translational motion in millimeters (*x*-, *y*-, *z*-) and rotational motion in degrees (pitch, roll, yaw). No participant had a certain range of motion that was >3 mm translational or 3 degrees rotational. Regression of covariates on signals from white matter and cerebrospinal fluid (excluding global signals). Band-pass filtering within 0.01–0.1 Hz was used to remove the effects of noise, such as breathing, heartbeat, and slow drift. The space was spatially transformed to standard stereotactic space in Montreal Neurological Institute (MNI) coordinates, resampled once at a resolution of 3 mm × 3 mm × 3 mm, and spatially smoothed using a 4-mm Gaussian kernel (FWHM) to reduce spatial noise before statistical analysis.

### 2.6 Seed-based FC analysis

Regions of interest (ROIs) defined in the Brainnetome atlas were used as the source of analysis to ensure the reliability of the ROIs. Four ROIs were identified based on previously validated cerebellar regions of interest (“seeds”). The radius of the spherical seeds was 5 mm, and was based on the bilateral cerebellum IX (left lobule IX: −7, −54, −50; right cerebellum IX: 8, −55, −48), bilateral cerebellum VI (left cerebellum VI: −14, −69, −22; right cerebellum VI: 18, −68, −23), left cerebellum Crus I/II (left cerebellum Crus I: −42, − 65, −33; left cerebellum Crus II: −17, −81, −41), right cerebellum Crus I/II (right cerebellum Crus I: 42, −66, −32; right cerebellum Crus II: 17, −81, −39) centered at the peak MNI coordinates, respectively. The signal time of the mean time course of the spherical region for each ROI and the signal time of the other voxels were calculated separately. Pearson correlation coefficients between the sequences were used to create FC maps for each ROI. Normality was improved by converting the correlation coefficients to *z*-values using Fisher’s *r*-to-*z* transformation. *Z*-score FC plots within the stroke and HC groups were statistically analyzed using one-sample *t*-tests and corrected by Gaussian Random Fields (GRF) (voxel significance: *p* < 0.001, cluster significance: *p* < 0.05) to minimize areas with false positives. The medical professionals used the MRIcron software to draw the lesions for every patient. In addition, differences in functional connectivity were calculated between patients with left-lesion and right-lesion strokes (Supplementary methods).

### 2.7 Statistical analyses

All statistical analyses were performed using SPSS Version 26.0. Statistical significance was set at *p <* 0.05. Continuous variables and categorical variables were shown as either the mean (SD) or median (interquartile range) values. Two sample *t*-tests were used to compare data between two groups. The Wilcoxon test was used to compare two groups and discrete variables were calculated using Chi-square tests. Spearman correlation analysis was performed between the *Z* scores of cognitive function and abnormal FCs. We evaluated the association between cognitive function and abnormal FCs by further multiple linear analyses adjusting for age, years of education, and IADL score.

## 3 Results

### 3.1 Demographic information, clinical profile, and neuropsychological assessment

The results of the clinical assessment for all participants are shown in Table 2. A total of 24 patients had stroke lesions on the left side, 14 patients had stroke lesions on the right side, and 1 patient had stroke lesions on the bilateral sides (Supplementary Figure S1). There were no significant differences in age, sex, educational level, or scores for IQCODE, HAMD, and BADL between the stroke and HCs. The IADL score in the stroke group was significantly higher than that in the HC group (*p* < 0.001). The proportion of individuals with smoking or drinking, and hypertension in the stroke group was higher than those in the HC group (*p* < 0.001). The median (interquartile range) NIHSS score was 2.00 (3.00). The volume of lesions ranged from 213.57 to 23862.31 mm^3^, and the average volume of lesions was 4533.16 ± 4909.49 mm^3^ (Supplementary Table S1). The neuropsychological assessment of the participants is shown in Table 3. The stroke group showed a dramatically lower *Z*-score when compared to the HC group in MoCA-Beijing, as well as several cognitive domains, such as episodic memory, language, visuospatial ability, attention, and executive function (*P* < 0.05) (Table 3).

**Table 2:** Demographic information of control and stroke group.

	Control group (*n* = 29)	Stroke group (*n* = 39)	*p*-value
Age (years, mean ± SD)	51.07 ± 6.77	51.88 ± 10.09	0.705
Sex (male, %)	21 (72.4)	29 (80.6)	0.439
Education [years, median (IQR)]	11 (10 ~ 12)	10 (8 ~ 12)	0.411
NIHSS at admission (scores, mean ± SD)	–	2.67 ± 2.042	–
Functional status			
Modified Rankin Scale [scores, median (IQR)]	–	1.00 (1.00)	–
IQCODE (scores, mean ± SD)	3.05 ± 0.09	3.11 ± 0.13	0.052
HAMD [scores, median (IQR)]	1.00 (0.00–3.00)	2.00 (1.00 ~ 5.00)	0.069
Instrumental ADL [scores, median (IQR)]	8.00 (8.00–8.00)	8.00 (8.00–10.00)	<0.001**
Basic ADL (scores, mean ± SD)	6.00 ± 0.00	6.19 ± 0.75	0.168
Diameter of lesion (mm, mean ± SD)	–	17.23 ± 8.59	–
Volume of lesion (mm^3^, mean±SD)	–	4533.16 ± 4909.49	–
Left basal ganglia (cases, %)	–	29 (74.4)	–
Hypertension (cases, %)	2 (6.9)	23 (65.7)	<0.001**
Diabetes (cases, %)	0 (00)	11 (31.4)	0.27
Current or ever drinking (cases, %)	4 (13.8)	25 (71.4)	<0.001**
Current or ever smoking (cases, %)	3 (10.3)	23 (65.7)	<0.001**

NIHSS, National Institute of Health Stroke Scale; IQCODE, Informant Questionnaire on Cognitive Decline in the Elderly. HAMD, Hamilton Depression Rating Score; ADL, Activities of Daily Living Scale; *p < 0.05, **p < 0.01.

**Table 3:** Comparison of neuropsychological tests between healthy controls and stroke group.

	Healthy subjects	Stroke group	*P*-value
	(*n* = 29)	(*n* = 39)	
Global cognition			
MOCA [median (IQR), points]	0.56 (0.255 ~ 1.08)	−0.05(−0.88 ~ 0.36)	<0.001**
Episodic memory (mean ± SD)	1.203 ± 1.104	−0.811 ± 1.498	<0.001**
AVLT (mean ± SD)	0.601 ± 0.714	−0.404 ± 0.960	<0.001**
RCFT-DR (mean ± SD)	0.602 ± 0.715	−0.406 ± 0.959	<0.001**
Language[median (IQR)]	0.88 (0.33 ~ 1.705)	−0.56(−1.48 ~ 0.7)	<0.001**
AFT (mean ± SD)	0.520 ± 0.917	−0.410 ± 0.958	<0.001**
BNT [median (IQR)]	0.68 (0.32 ~ 0.86)	−0.22(−0.58 ~ 0.5)	<0.001**
Visuospatial ability [median (IQR)]	0.37 (0.305 ~ 0.49)	0.24(−0.14 ~ 0.37)	<0.001**
RCFT-copy [median (IQR)]	0.37 (0.305 ~ 0.49)	0.24(−0.14 ~ 0.37)	<0.001**
Attention [median (IQR)]	1.07 (0.615 ~ 1.89)	−0.67(−1.99 ~ 0.69)	<0.001**
SDMT (mean ± SD)	0.738 ± 0.671	−0.511 ± 0.861	<0.001**
TMT-A [median (IQR)]	−0.5(−0.9 ~ −2.05)	0.12(−0.41 ~ 1.07)	<0.001**
Executive function [median (IQR)]	0.15(−0.2 ~ 0.815)	0.27(−0.38 ~ 0.74)	<0.001**
TMT-B-TMT-A (mean ± SD)	−0.097 ± 0.764	0.081 ± 0.683	0.318
CWT-C [median (IQR)]	0.35 (0.35 ~ 0.35)	0.2 (0.05 ~ 0.35)	<0.001**

MoCA, Montreal Cognitive Assessment; AVLT, Auditory Verbal Learning Test; RCFT, Rey-Osterrieth Complex Figure Test; AFT, Animal Fluency Test; BNT, Boston Naming Test; SDMT, Symbol Digit Modalities Test; TMT-A, Chinese modified version of the Trail Making Test; CWT-C, Color-Word Test-Chinese version; *p < 0.05, **p < 0.01. Adjusted for age, education level and IADL.

### 3.2 Comparison of the FC between HC group and stroke group

#### 3.2.1 The FC between the bilateral cerebellum IX and other voxels in the whole brain

**Figure 1:**
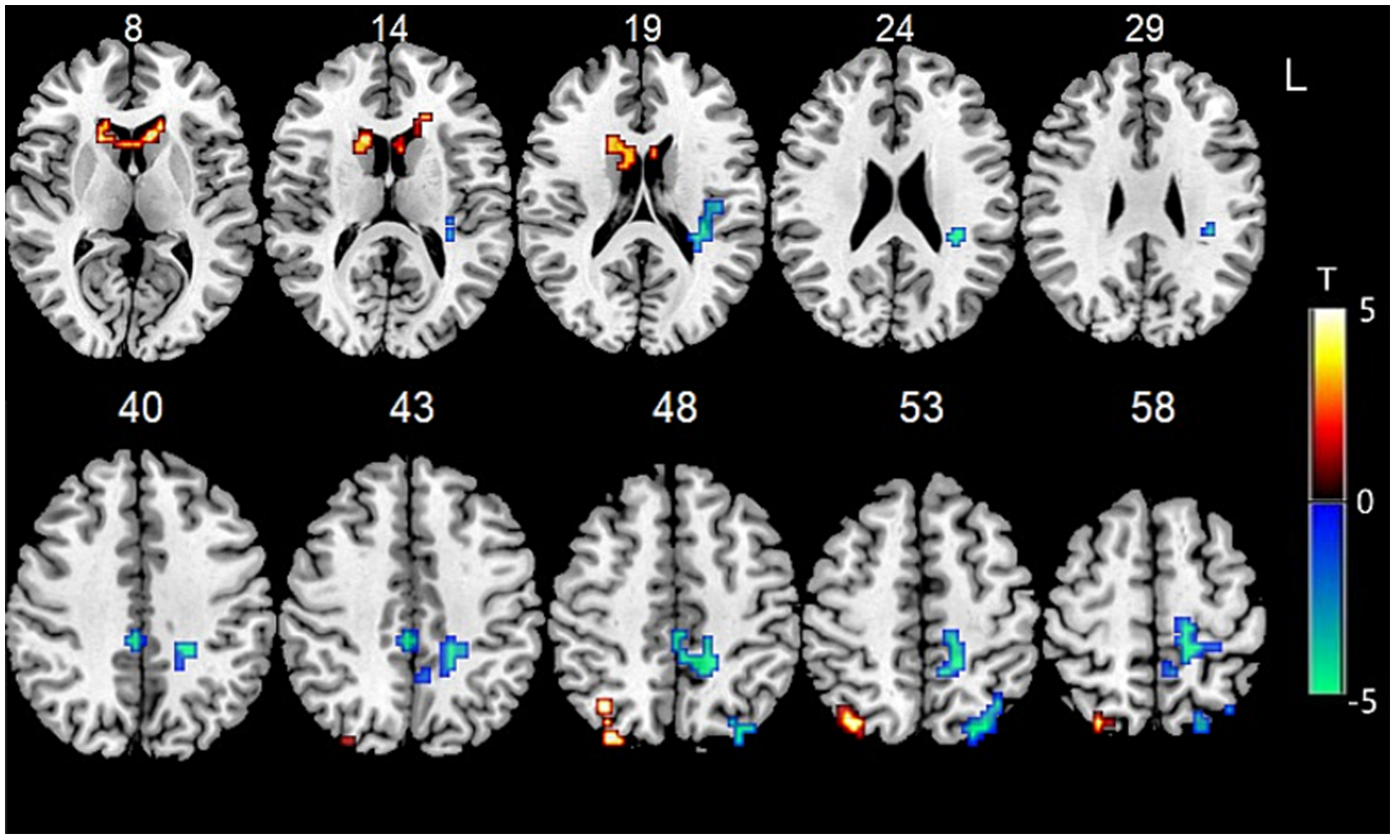
Functional connectivity between bilateral cerebellum IX and other voxels in the whole brain.

Data showed that the FC between the bilateral cerebellum IX and BG (especially the head of the caudate nucleus) were significantly increased, while the FC between bilateral cerebellum IX and the right caudal cuneus gyrus and the left occipital cortex were significantly decreased. The FC between the bilateral cerebellum IX, bilateral inferior occipital gyrus, and bilateral superior lateral occipital gyrus were also significantly decreased (Figure 1).

#### 3.2.2 The FC between the bilateral cerebellum VI and other voxels in the whole brain

**Figure 2:**
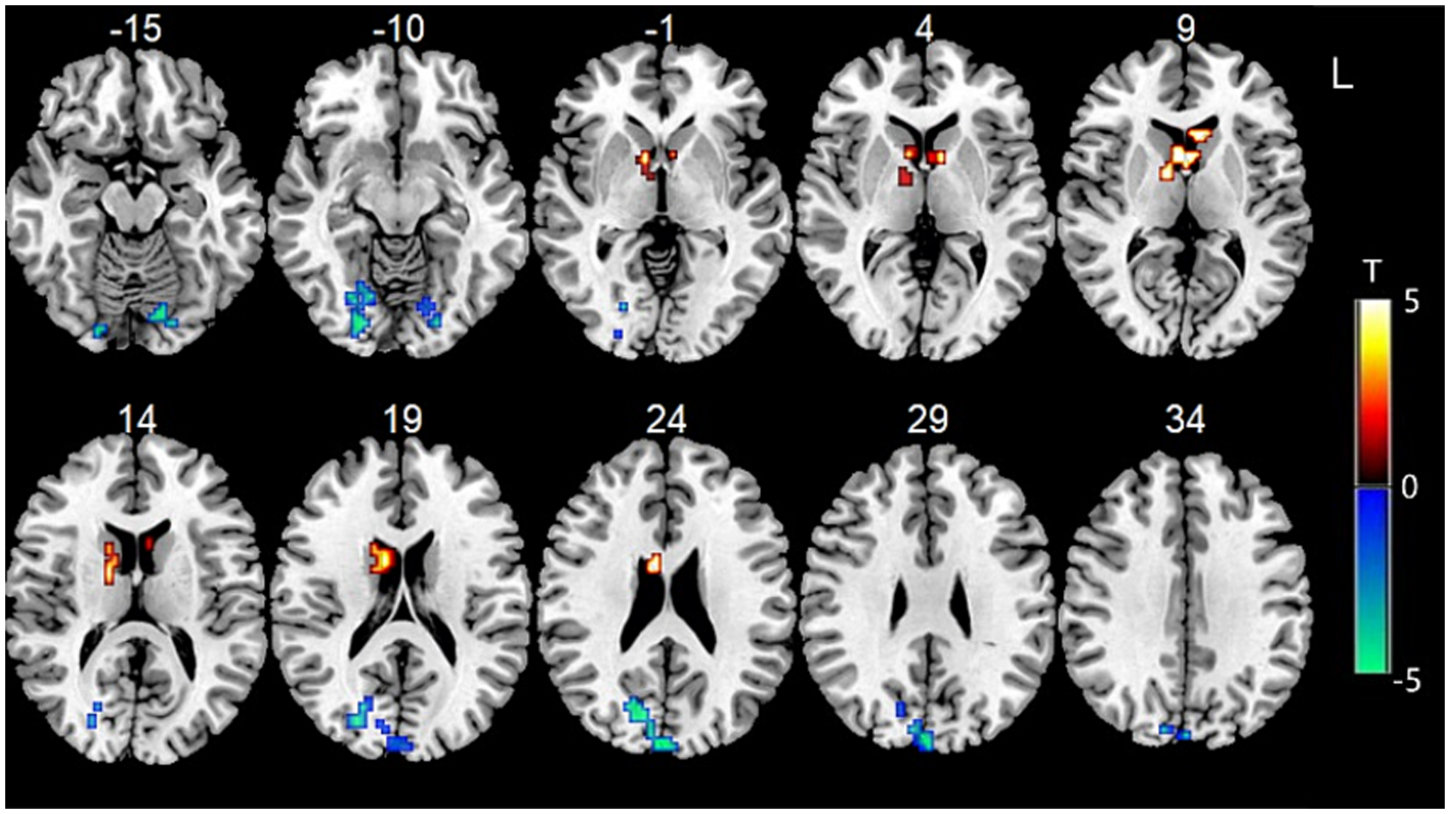
Functional connectivity between bilateral cerebellum VI and other voxels in the whole brain.

Data showed that the FC between bilateral cerebellum VI and the left anterior cuneus, left middle parafrontal lobule, left posterior central gyrus, right limbic cingulate, and left insula gyrus was significantly decreased. The FC between the bilateral cerebellum VI and the right dorsal caudate of the BG, the left ventral caudate of the BG, and the inferior parietal lobule were significantly increased (Figure 2).

#### 3.2.3 The FC between the left cerebellum Crus I/II and other voxels in the whole brain

**Figure 3:**
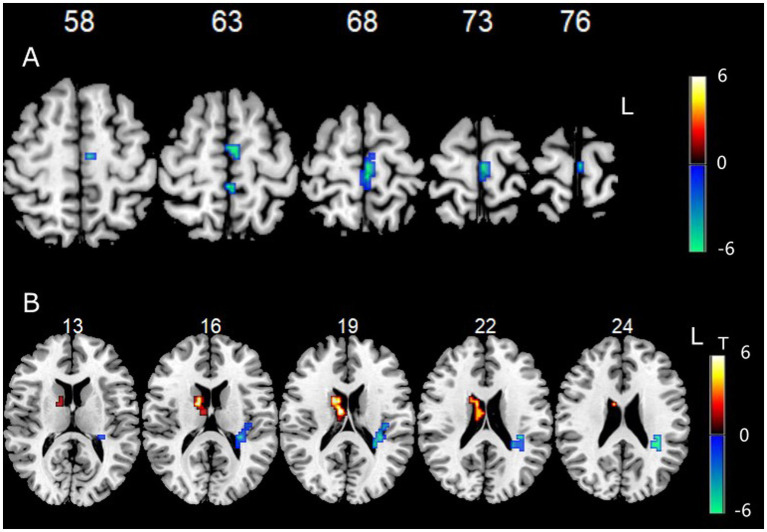
**(A)** Functional connectivity between left cerebellum Crus I/II and other voxels in the whole brain. **(B)** Functional connectivity between right cerebellum Crus I/II and other voxels in the whole brain.

Data showed that the FC between the left cerebellum Crus I/II and the left paracentral lobule, as well as the left superior frontal gyrus, were significantly decreased (Figure 3A).

#### 3.2.4 The FC between the right cerebellum Crus I/II and other voxels in the whole brain

Data showed that the FC between the right cerebellum Crus I/II and the right BG (especially the head of the caudate nucleus) was significantly increased, while the FC between the right cerebellum Crus I/II and the left insula and left sub parietal lobule were significantly decreased (Figure 3B).

### 3.3 Comparison of the FC between left-lesioned group and right-lesioned group

#### 3.3.1 The FC between the bilateral cerebellum IX and other voxels in the whole brain

Compared with the right-lesioned group (14 patients), the left-lesioned group (24 patients) showed significantly decreased FCs of cerebellum IX with the right inferior parietal lobule, right superior parietal lobule, right superior temporal gyrus, right middle temporal gyrus, and the left postcentral gyrus (*p* < 0.05, GRF-corrected). In addition, the FC between cerebellum IX and the right middle frontal gyrus was significantly increased (*p* < 0.05, GRF-corrected) (Supplementary Figure S2).

#### 3.3.2 The FC between the right cerebellum Crus I/II and other voxels in the whole brain

Compared with the right-lesioned group (14 patients), the left-lesioned group (24 patients) showed significantly decreased FC between the right cerebellum Crus I/II and the left postcentral gyrus (*p* < 0.05, GRF corrected) (Supplementary Figure S3).

### 3.4 Correlation analysis between FCs and cognitive function

**Figure 4:**
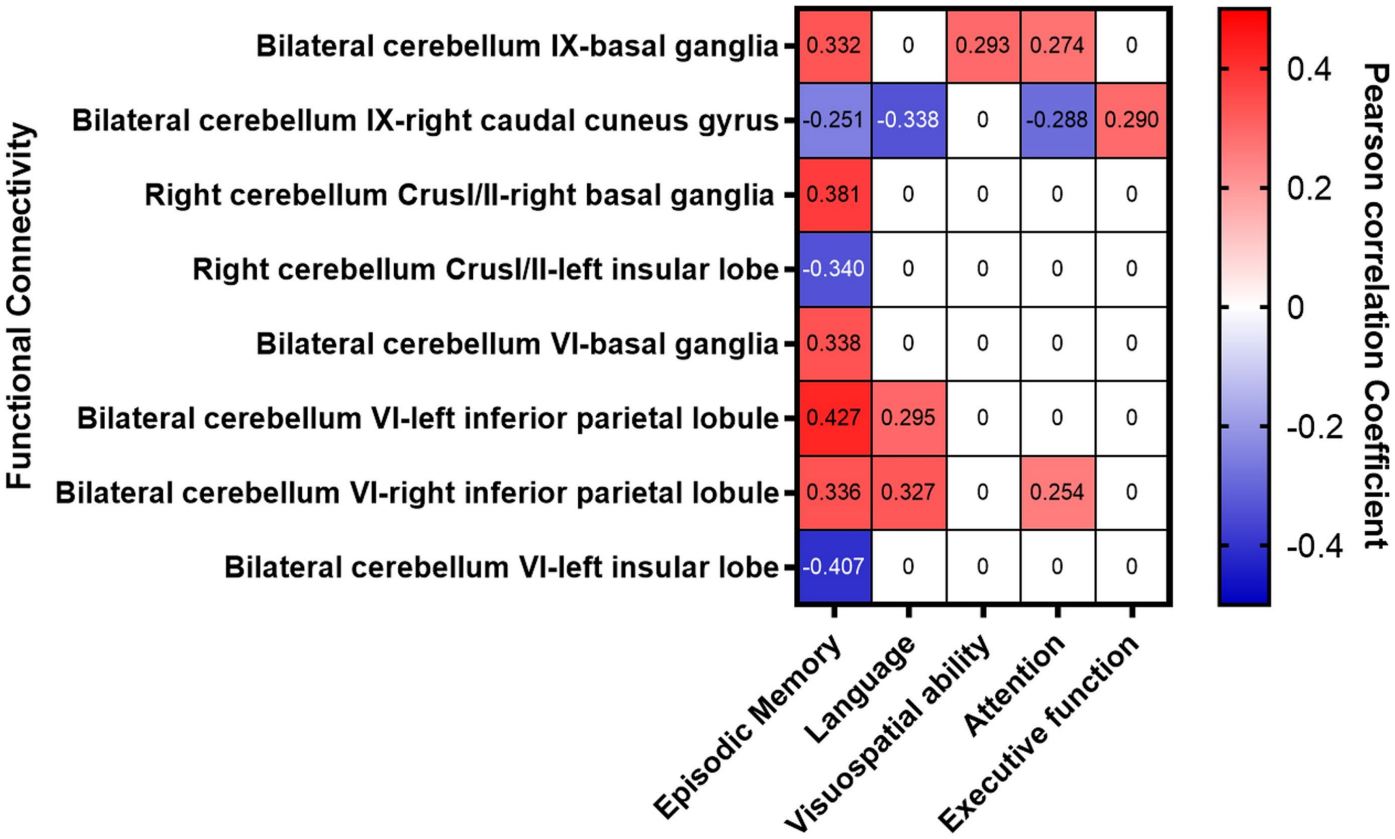
Correlation analysis between abnormal FCs and cognitive function.

The FC between the bilateral cerebellum IX and the BG (especially the head of the caudate nucleus) in stroke patients was significantly increased, which was significantly and positively correlated with the *Z* scores of episodic memory, visuospatial ability, and attention. The FCs between the right cerebellum Crus I/II and the right BG (especially the head of the caudate nucleus) were significantly increased, which was positively correlated with the *Z* score of episodic memory. The FC between the bilateral cerebellum VI and bilateral inferior parietal lobule was significantly increased which was significantly and positively correlated with episodic memory, language and attention. The FC between the bilateral cerebellum VI and left insular lobe was significantly decreased, which was significantly and negatively correlated with episodic memory (Figure 4 and Table 4).

**Table 4:** Association between abnormal FCs and cognitive function.

Correlations	Episodic Memory		Language		Visuospatial ability		Attention		Executive function	
	*r*	*P*-value	*r*	*P*-value	*r*	*P*-value	*r*	*P*-value	*r*	*P*-value
Bilateral cerebellum IX and basal ganglia	0.332	0.006^**^	–		0.293	0.017^*^	0.274	0.026^*^	–	
Bilateral cerebellum IX and right caudal cuneus gyrus	−0.251	0.042^*^	−0.338	0.006^**^	–		−0.288	0.019^*^	0.29	0.018^*^
Right cerebellum CrusI/II and right basal ganglia	0.381	0.002^**^	–		–		–		–	
Right cerebellum CrusI/II and left insular lobe	−0.34	0.005^**^	–		–		–		–	
Bilateral cerebellum VI and basal ganglia	0.338	0.006^**^	–		–		–		–	
Bilateral cerebellum VI and left inferior parietal lobule	0.427	0.001^**^	0.295	0.016^*^	–		–		–	
Bilateral cerebellum VI and right inferior parietal lobule	0.336	0.006^**^	0.327	0.007^**^	–		0.254	0.04^*^	–	
Bilateral cerebellum VI and left insular lobe	−0.407	0.001^**^	–		–		–		–	

*p < 0.05, **p < 0.01.

The FC between the bilateral cerebellum IX and the right caudal cuneus gyrus was significantly decreased, which was significantly and negatively correlated with the Z scores for memory, language, attention, and executive function. The FCs between the right cerebellum Crus I/II and the left insular lobe were decreased, and were significantly and negatively correlated with *Z* scores for episodic memory (Figure 4 and Table 4) (*p* < 0.017 after Bonferroni correction).

The difference of cerebellar-cortical FCs between the two groups (the left-lesioned group and the right-lesioned group) were not correlated with cognitive impairment.

## 4 Discussion

This work presented a comprehensive connectivity map between the cerebellum and cerebral cortex in acute ischemic stroke with BG infarcts and their association with cognition. In this study, we compared the changes in FC between the cerebellum and cortex in controls and stroke patients and explored their correlation with cognitive changes. Compared to the HC group, the stroke group showed decreased FCs in the cerebellum and the right caudal cuneus gyrus and left insular lobe, and enhanced FCs in the cerebellum with BG (especially the head of caudate nucleus) and inferior parietal lobule. The altered FCs were related to cognitive recovery in different domains. The altered cerebellar FCs indicated that the disruption and recombination of cerebello-cerebral networks occurred simultaneously. The enhanced FC between the cerebellum and BG (especially the head of caudate nucleus) may be a compensatory response for cognitive decline after an ischemic stroke with BG infarcts. Therefore, the cerebellum might be a new target of intervention for PSCI.

The cerebellar subregions were closely correlated with multiple cortical neural networks and associated with multiple cognitive domains. Several studies have shown that patients with cerebellar stroke performed worse on visuospatial tests (Botez-Marquard et al., 1994; Richter et al., 2007). Visuospatial ability is measured by the capacity to understand and identify the visual and spatial relationships among objects. Another study has noted that patients with lesions of the left cerebellum were more prone to having visuospatial deficits (Kellermann et al., 2012). The cerebellum IX has been reported to be involved in cognitive networks (Toniolo et al., 2020), particularly the DMN (Zheng et al., 2017). Multiple system atrophy (MSA) patients with impaired cognition showed decreased FC between the right cerebellum IX and bilateral cuneus, and was associated with the MoCA score (Li et al., 2023). The visuospatial deficits might be related to cerebellar-basal ganglia-cortical diaschisis. The BG is involved in motor function and cognition and has a close correlation with the cortex and cerebellum (Choi et al., 2012). In this study, stroke patients with BG infarcts were found to have enhanced FC between the bilateral cerebellum IX and BG, especially the head of caudate nucleus, and positively correlated with visuospatial ability, episodic memory and attention, which was consistent with the previous study. The head of the caudate nucleus is directly associated with the oculomotor and associative loops, which receive input from the cognitive areas, such as DLPFC, rostral anterior cingulate, and inferior frontal gyri (Parent and Hazrati, 1995). The DLPFC was involved in episodic memory, attention and executive function (Grahn et al., 2008). We hypothesized that the enhanced FC between the cerebellum and BG (especially the head of caudate nucleus) would help to maintain cognitive performance, which indicated that the cerebellum played a compensatory role in cognitive recovery after stroke with BG infarct.

The cerebellum is structurally connected to the cerebral cortex and is also known to have functional connectivity with the cerebral cortex that is related to a variety of cognitive functions, such as episodic memory (Hautzel et al., 2009). The cerebellum receives inputs and projections back to the cerebral cortex and previous studies have noted memory decline in patients with cerebellar disorders (Mariën et al., 2017). Prior researchers have demonstrated that the cerebellum Crus II and IX are related to the DMN in patients with vascular cognitive impairment (Toniolo et al., 2020). The DMN generates episodical memory (Buckner et al., 2008) and our study found that the FC between the bilateral cerebellum IX and right caudal cuneus gyrus was decreased and negatively correlated with the *z* score for episodic memory. The FC between the right cerebellum IX and BG (especially the head of caudate nucleus) was enhanced and positively correlated with the *z* score for episodic memory. Another study reported that the changes in the posterior lateral cerebellum, primarily involving the Crus I/II, have previously been reported to be related to episodic memory (Castellazzi et al., 2018). This study found that the FC between the right cerebellum Crus I/II and the salience network (left insular lobe) were decreased and negatively correlated with the *z* score for episodic memory. The FC between the bilateral cerebellum VI and the salience network (left insular lobe) was decreased and negatively correlated with the *z* score for episodic memory. The FC between the bilateral cerebellum VI and the DMN (bilateral inferior parietal lobule) was enhanced and positively correlated with the *z* score for episodic memory. This indicated that several cerebellar subregions are involved in the memory process and these abnormal FCs might be activated during memory-related tasks.

Executive impairment and attention were found to be the most frequently impaired in cerebellar-involved cognitive dysfunction (Tedesco et al., 2011; Craig et al., 2021). Executive function is defined as the ability to plan, abstract, and task management, as well as the ability to maintain attention for long periods. The cerebellar projections to the frontoparietal cortex and its circuit may serve as the neural basis for the involvement of the cerebellum in executive functioning. This study found that the FC between the bilateral cerebellum IX and right caudal cuneus gurus was decreased and negatively correlated with *z* scores for attention and executive function. The cerebellum IX was reported to be strongly associated with the DMN network (Chanraud et al., 2011) which has been suggested to play a crucial part in the executive system (Christoff et al., 2009), and is also correlated with executive function and attention. This indicates that the cerebello-cerebral circuits were disrupted in BG stroke patients and were related to attention and executive dysfunction. In this study, we found that the FC between the bilateral cerebellum IX and BG (especially the head of caudate nucleus) was enhanced and positively correlated with the *z* score for attention. Several studies have suggested that the cerebellar-basal ganglia circuit is associated with executive function and attention (Habas, 2021). The FC between the cerebellum and BG is enhanced during the learning process (Sami et al., 2014) and the associations between cognitive tests and the cerebellar FCs with the BG and cerebral cortex were involved in episodic memory, and visuospatial ability attention, which are mostly presented in PSCI patients (Acharya et al., 2022). The FC between the bilateral cerebellum VI and the default mode network (bilateral inferior parietal lobule) was significantly and positively correlated with the *z* score for episodic memory, language, and attention. A study has identified that the cerebellum VI contributed to several intrinsic cortical networks, including the CEN, DMN, and SN (Sang et al., 2012). While the DMN and SN are well-known to be involved in language and attention (Seeley et al., 2007), the above networks may be the important cortico-cerebellar circuits. The increased FC between the cerebellum and BG (especially the head of caudate nucleus) might be a compensatory mechanism for cognition after a stroke.

As well as the domains of visuospatial ability, attention, executive function, and memory, the involvement of the cerebellum in language has also been determined. The cerebellar damage usually affects speech and is commonly called ataxic dysarthria. It is usually caused by the incoordination of vocal organs. Patients with cerebellar infarction also presented with dysfunction of verbal fluency and semantic access (Mariën et al., 2009). The underlying mechanism might be the disconnection between the cerebellum and frontal cortex. The Crus I/II and IX were found to be associated with language (Stoodley et al., 2016). Here, we found that the FC of the bilateral cerebellum IX and right caudal cuneus gyrus were decreased and negatively correlated with the *z* score for language. One recent study recruited 22 patients with chronic cerebellar stroke and revealed that semantic fluency was severely impaired when there was damage to the right VI and Crus I/II (Chirino-Pérez et al., 2022). The 5 Hz rTMS of the bilateral cerebellum significantly improves multi-domain cognitive functions, such as memory, attention, and language in patients with Alzheimer’s disease (Yao et al., 2022). These results suggested that different cerebellar subregions showed abnormal FC with multiple cortical areas and was associated with cognitive function and might be compensatory for cognitive decline after stroke. The cerebrocerebellar circuit has been thought to be the mechanism involved in cerebellum-related cognition.

Furtherly, the present study explored the significant differences in cerebellar-cortical FC between the left-side lesion group (24 patients) and the right-side lesion group (14 patients). The FC between cerebellum IX and the right parietal lobe (inferior parietal lobule, superior parietal lobule), right temporal lobe (superior temporal gyrus, middle temporal gyrus), and left postcentral gyrus was significantly decreased in the left-side lesion group, whereas FC was stronger in the right-side lesion group, which may reflect a more extensive effect of the left-side lesion on the transhemispheric network. This phenomenon is consistent with the dominant role of the left side of the brain in cognition, language and sensorimotor functions (Stockert et al., 2020). At the same time, FC between cerebellum IX and the right middle frontal gyrus was significantly increased in the left-side lesion group, suggesting that patients may have compensated for the loss of cognitive function after stroke by strengthening cerebellar-frontal collaboration. This compensatory enhancement suggested that the cerebellum may have an important role in functional remodeling after stroke (Ntakou et al., 2022). In addition, the FC between the right cerebellum Crus I/II and the left postcentral gyrus was significantly decreased in the left-lesioned group, suggesting impaired modulation of the transhemispheric sensorimotor network, which may be associated with motor dysfunction in patients (Calautti and Baron, 2003). Although the present study revealed significant differences in cerebellar-cortical FC between the left-and right-lesioned groups, further correlation analyses did not reveal significant associations between these FC differences and cognitive assessment (Supplementary Table S2). This result may be due to the small sample size and insufficient statistical efficacy to detect potentially weak correlations. Future studies should expand the sample size to improve the reliability of the results.

There are several limitations to this study. Firstly, the sample size was only 68, which is relatively small, further research with a large cohort should be conducted to replicate our current findings. Furthermore, this study does offer a theoretical or imaging basis for early intervention of PSCI. For example, rTMS stimulation of the cerebellum through the cerebello-cerebral circuits might delay the cognitive decline in ischemic stroke with BG infarcts. Secondly, this study did not address the specific associations between FC (cerebellum and cortex) and cognition in different side-lesion strokes (left or right basal ganglia infarcts), due to the small sample size, which may provide a more comprehensive understanding. More studies designed to explore the impact of lesion laterality on the PSCI outcomes are needed in the future, and this may help to understand the mechanisms of cerebellum on BG infarcts. Thirdly, we were not able to identify the pre-stroke condition or apolipoprotein E (APOE) genotype of the patients, which might have a potential impact on cognition, though we screened the pre-stroke cognitive conditions using IQCODE. Therefore, we will collect APOE genotypes from stroke patients in the future. Fourthly, this study was cross-sectional, and causal relationships could not be established and cognitive status dynamically changes over time, therefore, future studies should obtain this follow-up information, and investigate the relationship between altered FCs and cognitive changes.

## 5 Conclusion

We have compared for the first time, the FC between the cerebellum and cortex in ischemic stroke patients with BG infarcts and controls, and found disruption of the cerebro-cerebellar circuits, and also neural repairment at the same time. This interconnection between the cerebellum IX, VI, and Crus I/II is associated with episodic memory, visuospatial ability, language, executive function, and attention. The cerebellum plays a compensatory role in PSCI with BG infarcts and we hypothesized that the specific FCs between the cerebellum and cerebral cortex might have high potential as therapeutic targets for cognitive recovery and might provide a theoretical basis for cognitive modulation. The non-invasive brain stimulation for the cerebellum, such as transcranial direct current stimulation and repetitive transcranial magnetic stimulation, may improve cognition, and targeting to stimulate narrow subregions of the cerebellum produces positive effects on cognitive tasks such as executive function, speech, episodic memory, and visuospatial ability. In addition, cerebellum-targeted rehabilitation will provide new insights for interventional PSCI.

## Data Availability

The raw data supporting the conclusions of this article will be made available by the authors, without undue reservation.

## Footnotes

^1^ http://rfmri.org/dpabi

## Supplementary material

SUPPLEMENTARY FIGURE S1 Patient lesion distribution.

SUPPLEMENTARY FIGURE S2 The different functional connectivity between the bilateral cerebellum IX and cortex in left-lesioned group and right-lesioned group.

SUPPLEMENTARY FIGURE S3 The different functional connectivity between right cerebellum Crus I/II and cortex in left-lesioned group and right-lesioned group.
