# Supplementary figures and images for: Cerebellar-cerebral circuits functional connectivity in patients with cognitive impairment after basal ganglia stroke: a pilot study
Source: Front Aging Neurosci. 2025 Jan 30;17:1478891. doi: 10.3389/fnagi.2025.1478891 (PMC11821925; doi:10.3389/fnagi.2025.1478891)

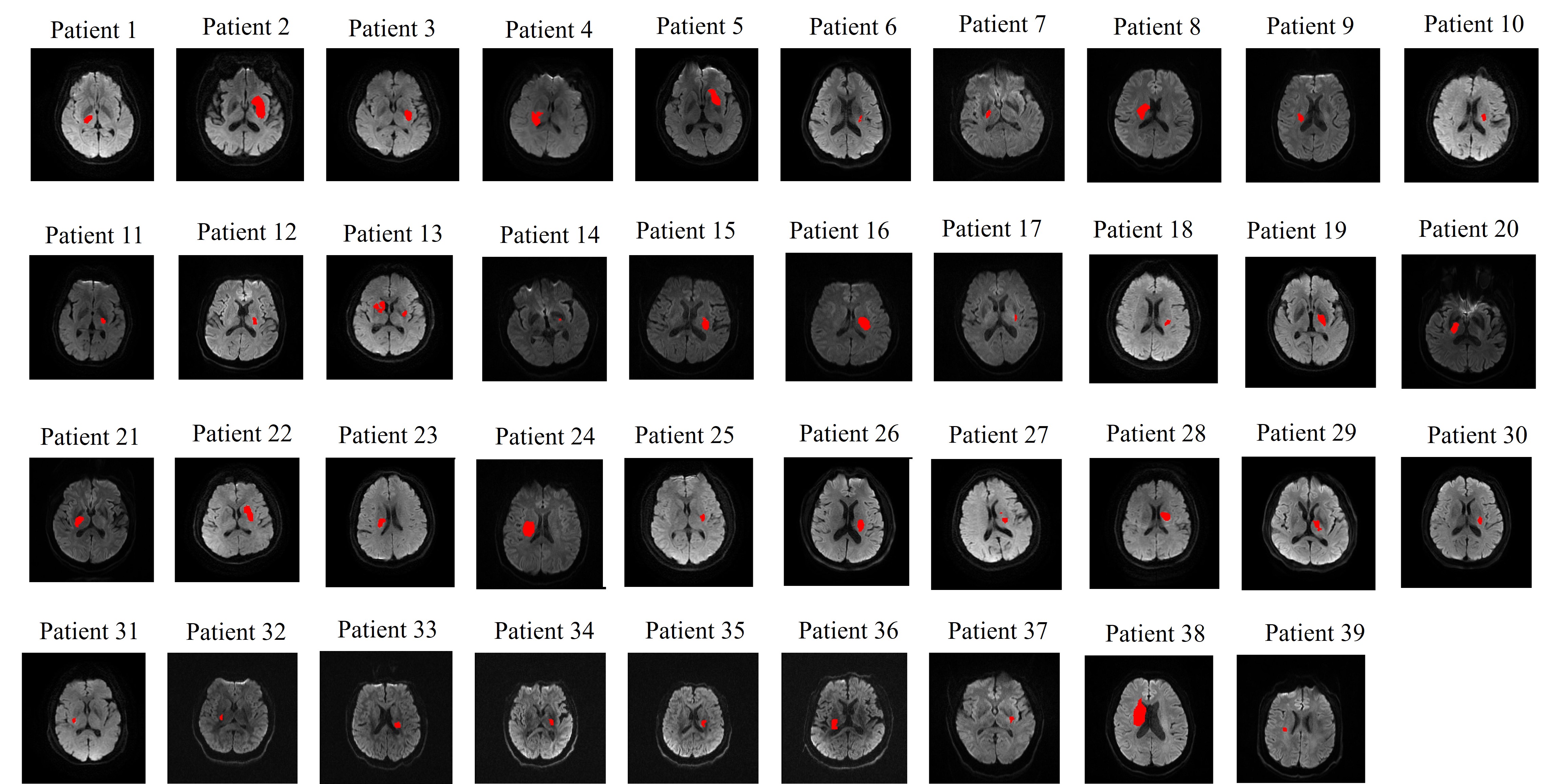

Supplement: SUPPLEMENTARY FIGURE S1 — Patient lesion distribution. [file Image_1.JPEG]

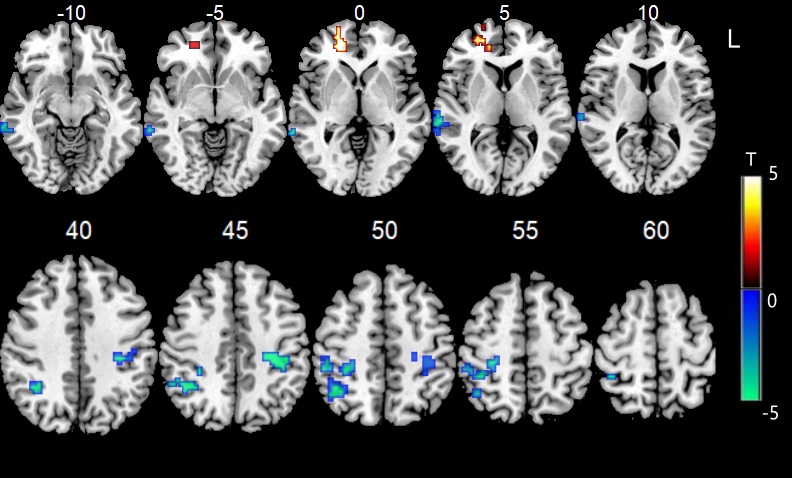

Supplement: SUPPLEMENTARY FIGURE S2 — The different functional connectivity between the bilateral cerebellum IX and cortex in left-lesioned group and right-lesioned group. [file Image_2.JPEG]

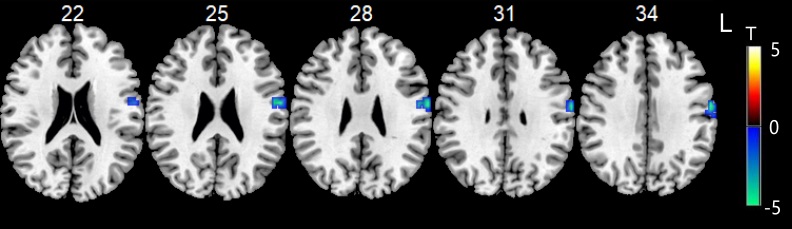

Supplement: SUPPLEMENTARY FIGURE S3 — The different functional connectivity between right cerebellum Crus I/II and cortex in left-lesioned group and right-lesioned group. [file Image_3.JPEG]
