# Supplementary material for: Cerebellar-cerebral circuits functional connectivity in patients with cognitive impairment after basal ganglia stroke: a pilot study
Source: Front Aging Neurosci. 2025 Jan 30;17:1478891. doi: 10.3389/fnagi.2025.1478891 (PMC11821925; doi:10.3389/fnagi.2025.1478891)
Supplement: Supplementary file 4 [file Table_1.docx]

**Supplement
Supplemental methods** To further explore the differences in functional connectivity between patients with left- versus right-sided lesion stroke, we divided the patients into two groups: a left-sided lesion group (24 patients) and a right-sided lesion group (14 patients). Based on the cerebellar seed points used in the original analysis (cerebellum IX, left cerebellum Crus I/II, right cerebellum Crus I/II, and cerebellum VI), pearson correlation coefficients were calculated separately for each region of interest versus the other voxel sequences to create FC maps, and normality was improved by converting the correlation coefficients to z-values using Fisher's r-to-z transformation. Z-score FC plots within the stroke and HC groups were statistically analyzed using independent samples t-tests and corrected using Gaussian Random Fields (GRF) (voxel significance: p < 0.001, cluster significance: p < 0.05).

**Supplemental results**

Supplemental table S1. volumes of lesion for each patient

| Patient | Volumes of lesion (mm^3^) |
| --- | --- |
| 1 | 1930.115 |
| 2 | 23862.3 |
| 3 | 8722.266 |
| 4 | 4363.337 |
| 5 | 13246.88 |
| 6 | 696.0938 |
| 7 | 1062.032 |
| 8 | 5978.945 |
| 9 | 1473.749 |
| 10 | 1070.508 |
| 11 | 864.8229 |
| 12 | 2089.366 |
| 13 | 11659.57 |
| 14 | 305.8594 |
| 15 | 5369.822 |
| 16 | 6861.44 |
| 17 | 2088.264 |
| 18 | 479.8828 |
| 19 | 4202.93 |
| 20 | 3758.994 |
| 21 | 3143.377 |
| 22 | 11005.66 |
| 23 | 2842.383 |
| 24 | 9331.558 |
| 25 | 2921.302 |
| 26 | 6507.422 |
| 27 | 806.8359 |
| 28 | 3833.789 |
| 29 | 2250.879 |
| 30 | 2768.555 |
| 31 | 3127.148 |
| 32 | 552 |
| 33 | 2392 |
| 34 | 213.5654 |
| 35 | 1524.081 |
| 36 | 4288 |
| 37 | 1587.081 |
| 38 | 15138.58 |
| 39 | 2471.795 |

Supplemental table S2. Correlations of abnormal cerebellar-cortical FCs between the left-and right-lesioned groups with cognitive function

| Correlations | Episodic Memory | | Language | | Visuospatial ability | | Attention | | Executive function | |
| --- | --- | --- | --- | --- | --- | --- | --- | --- | --- | --- |
|  | r | P value | r | P value | r | P value | r | P value | r | P value |
| Bilateral cerebellum IX  and right inferior parietal lobule | -0.215 | 0.208 | -0.122 | 0.478 | 0.014 | 0.937 | -0.192 | 0.261 | 0.098 | 0.566 |
| Bilateral cerebellum IX  and right superior parietal lobule | -0.245 | 0.149 | -0.248 | 0.149 | 0.055 | 0.750 | -0.122 | 0.480 | -0.019 | 0.912 |
| Bilateral cerebellum IX  and right superior/ middle temporal gyrus | -0.151 | 0.379 | -0.209 | 0.222 | 0.065 | 0.707 | -0.068 | 0.693 | 0.016 | 0.925 |
| Bilateral cerebellum IX  and left postcentral gyrus | -0.190 | 0.268 | -0.154 | 0.371 | 0.013 | 0.941 | -0.027 | 0.876 | 0.030 | 0.863 |
| Bilateral cerebellum IX  and right middle frontal gyrus | 0.234 | 0.170 | 0.085 | 0.620 | 0.097 | 0.575 | -0.160 | 0.351 | -0.014 | 0.935 |
| right Cerebellum Crus I/II and left postcentral gyrus | -0.215 | 0.209 | -0.191 | 0.265 | 0.007 | 0.969 | -0.024 | 0.889 | -0.105 | 0.541 |
